# Mechanism of action and therapeutic efficacy of Aurora kinase B inhibition in MYC overexpressing medulloblastoma

**DOI:** 10.18632/oncotarget.3245

**Published:** 2014-12-31

**Authors:** Roberto Jose Diaz, Brian Golbourn, Claudia Faria, Daniel Picard, David Shih, Denis Raynaud, Michael Leadly, Danielle MacKenzie, Melissa Bryant, Matthew Bebenek, Christian A. Smith, Michael D. Taylor, Annie Huang, James T. Rutka

**Affiliations:** ^1^ The Hospital for Sick Children. Arthur and Sonia Labatt Brain Tumour Research Centre, Toronto, Ontario, Canada; ^2^ Department of Laboratory Medicine & Pathobiology, University of Toronto, Toronto, Ontario, Canada; ^3^ Division of Neurosurgery, Department of Surgery, University of Toronto, Toronto, Ontario, Canada; ^4^ Analytical Facility for Bioactive Molecules, The Hospital for Sick Children, Toronto, Ontario, Canada

**Keywords:** Aurora kinase, medulloblastoma, tumor biology, molecular therapy, cell-cycle

## Abstract

Medulloblastoma comprises four molecular subgroups of which Group 3 medulloblastoma is characterized by *MYC* amplification and MYC overexpression. Lymphoma cells expressing high levels of MYC are susceptible to apoptosis following treatment with inhibitors of mitosis. One of the key regulatory kinases involved in multiple stages of mitosis is Aurora kinase B. We hypothesized that medulloblastoma cells that overexpress MYC would be uniquely sensitized to the apoptotic effects of Aurora B inhibition. The specific inhibition of Aurora kinase B was achieved in MYC-overexpressing medulloblastoma cells with AZD1152-HQPA. MYC overexpression sensitized medulloblastoma cells to cell death upon Aurora B inhibition. This process was found to be independent of endoreplication. Using both flank and intracranial cerebellar xenografts we demonstrate that tumors formed from MYC-overexpressing medulloblastoma cells show a response to Aurora B inhibition including growth impairment and apoptosis induction. Lastly, we show the distribution of AZD1152-HQPA within the mouse brain and the ability to inhibit intracranial tumor growth and prolong survival in mice bearing tumors formed from MYC-overexpressing medulloblastoma cells. Our results suggest the potential for therapeutic application of Aurora kinase B inhibitors in the treatment of Group 3 medulloblastoma.

## INTRODUCTION

Recent genetic analysis of medulloblastoma (MB) has revealed four genetically and epidemiologically distinct subgroups.[1, 2] One of these subgroups designated as Group 3 medulloblastoma (G3MB) affects primarily infants and children and carries an overall survival of 45% for infants and 58% for children at five years as reported in a retrospective international meta-analysis.[3] Given the poor clinical outcomes observed in patients with G3MB tumors with current treatment regimens, there is a need to explore novel therapeutic options. We have recently reported on the in-vitro pro-apoptotic and in-vivo cytostatic effects of Aurora B inhibition in glioblastoma using the inhibitor AZD1152-HQPA.[4] Aurora kinase B (Aurora B) is an essential serine/threonine kinase responsible for regulation of multiple events in mitosis including chromosome condensation, centromere-kinetochore dynamics, chromatid segregation, and cytokinesis.[5] One of the hallmarks of Aurora kinase B inhibition is G2/M arrest and subsequent escape into endoreplication cycles.[6] Multiple regulatory or structural components essential to mitotic progression have been previously targeted to block tumor cell proliferation including microtubules,[7] cyclin dependent kinase 1,[8] Aurora kinases,[9] and Polo-like kinase 1.[10-12] When tumor cells overexpress c-Myc (MYC) either endogenously or by genetic modification, the induction of a G2/M arrest has been shown to trigger tumor cell death in-vitro and to block tumor growth in-vivo.[8, 13, 14] This observation was also made recently in B-cell and T-cell lymphoma cells overexpressing MYC and treated with the Aurora B inhibitor AZD1152-HQPA.[15, 16] MYC expression has also been reported to confer sensitivity to radiation and DNA damaging agents (etoposide, cisplatin) in medulloblastoma cells.[17]

Amplification of *MYC*, as well as *MYC* overexpression, is a negative prognostic factor for overall survival in MB.[18, 19] Approximately 11% of G3MB tumors demonstrate *MYC* amplification.[20] Furthermore, all G3MB tumors express *MYC* at high levels and express genes associated with elevated MYC levels.[20] We hypothesized that MB cells overexpressing MYC would be uniquely sensitized to the effects of Aurora B inhibition and that this property could be harnessed for the *in-vivo* treatment of MYC-overexpressing MB tumors. The goal of our study was not only to determine if MYC overexpression in human MB cells sensitized the cells to the apoptotic effects of Aurora B inhibition, but also to further define the mechanism triggering this response. We demonstrate that Aurora B inhibition triggers cell death independent of DNA replication and that transient Aurora B inhibition results in a unique impaired growth response in MYC-overexpressing cells. Having defined the response time-course we proceeded to optimize *in-vivo* therapy with AZD-1152 HQPA, achieving a prolongation in survival of mice bearing cerebellar xenografts of MB cells having *MYC* amplification and endogenously overexpressing MYC.

## RESULTS

### Co-expression of Aurora B and MYC in Group 3 medulloblastoma

**Figure 1:**
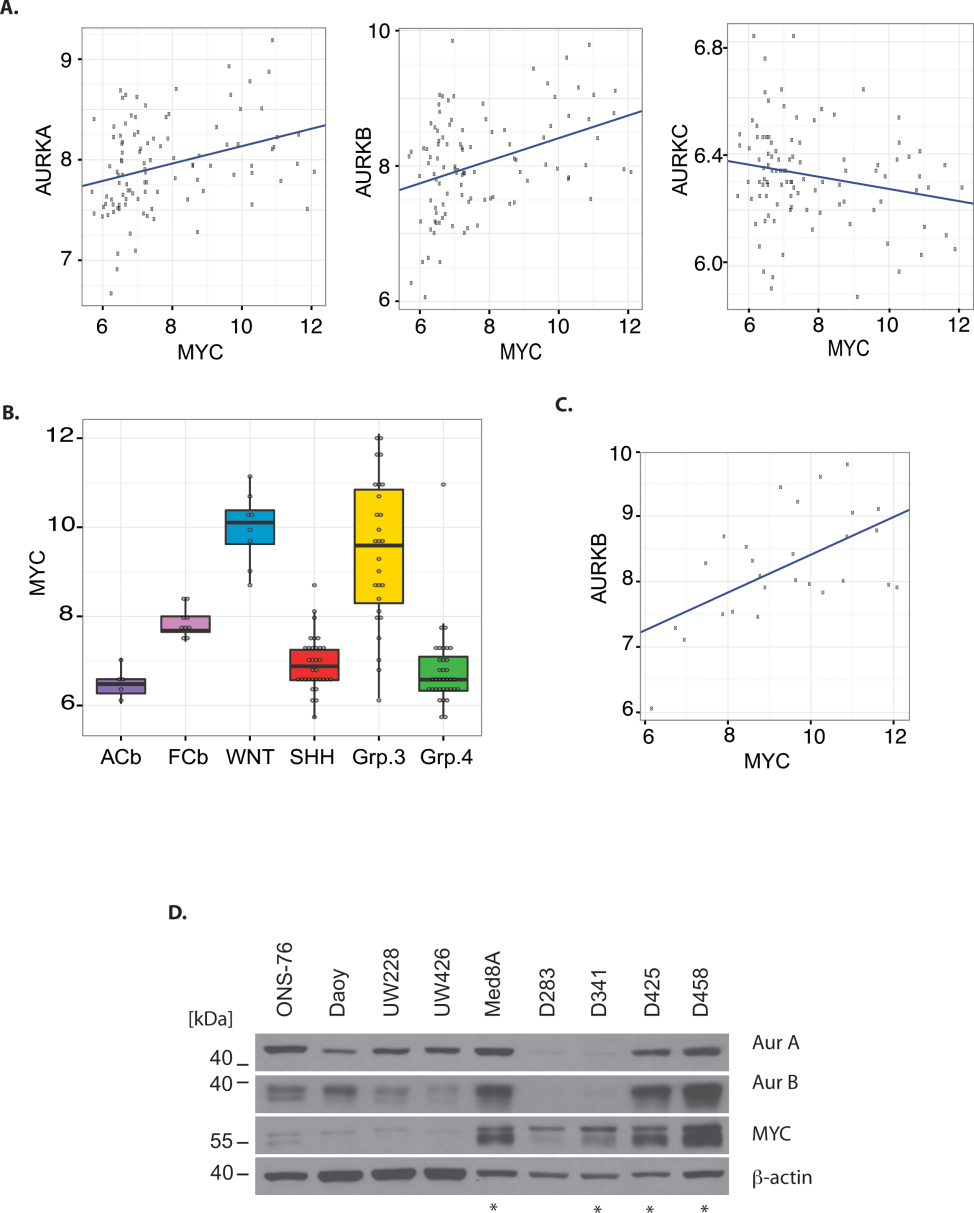
Aurora kinase mRNA and protein expression in relation to Myc expression in medulloblastoma A) mRNA expression of *AURKA*, *AURKB*, and *AURKC* in relation to *MYC* mRNA level in 103 medulloblastoma tumor samples. B) *MYC* mRNA expression in fetal cerebellum (fCb), adult cerebellum (aCb), and medulloblastoma tumors subgrouped according to RNA expression profile, ANOVA P<0.0001. C) Correlation between *AURKB* mRNA expression and MYC mRNA expression in medulloblastoma tumors subgrouped as Group 3. D) Western blot showing protein expression of Aurora A, Aurora B, and MYC in multiple medulloblastoma cell lines. Cell lines harboring *MYC* amplification are indicated by a star. The loading control was β-Actin. Total protein loaded was 30 μg.

MYC has been shown to directly regulate the expression of Aurora A and indirectly the expression of Aurora B in B-cell lymphoma.[15] Therefore, we sought to determine if Aurora kinase gene expression correlates with *MYC* expression in human MB. *AURKA* and *AURKB* mRNA expression showed a positive correlation with *MYC* mRNA expression (*AURKA* vs *MYC*: R=0.32, P=0.001, N=103; *AURKB* vs *MYC*: R=0.37, P = 0.0001, N=103) while no correlation exists between *AURKC* and *MYC* expression (Fig. 1A). The highest *MYC* expression was observed in WNT and G3MB relative to other subgroups, normal fetal cerebellum, and adult cerebellum (Fig. 1B). Furthermore, there was a modest correlation between *MYC* expression and Aurora B expression in G3MB (R=0.57, P=0.002, N=27, Fig. 1C). Although WNT tumors express high levels of *AURKB* mRNA we did not observe a correlation to *MYC* mRNA expression in this small subset of tumor samples (R=0.42, P=0.3, N=8). Aurora kinase gene expression is increased in fetal cerebellum and in all subgroups of MB compared to adult cerebellum, reflecting the proliferative capacity of fetal and tumor tissue.

To further evaluate the expression of Aurora kinase A and B in relation to MYC, protein expression in a number of unsynchronized MB cell lines was evaluated (Fig. 1D). The D425, D458 and MED8A cells, all of which have known amplification of *MYC*,[18, 21, 22] showed concurrent marked expression of both Aurora B and MYC protein. Using a NanoString assay with a probe set for MB subgroup signature genes,[23] the D425 cell line was demonstrated to share a similar gene expression signature as G3MB tumors (Supplementary Fig. S1). The D425 cell line was derived from primary tumor tissue,[21] whereas the D458 line was derived from cerebrospinal fluid within the same patient after failure of radiotherapy and chemotherapy with cyclophosphamide, cisplatin, and vincristine.[22, 24] As such, our results suggest that the concordance between Aurora B and MYC expression is maintained in the setting of recurrent G3MB.

### Myc overexpression sensitizes medulloblastoma cells to cell death induced by Aurora B inhibition

The UW228 and UW426 MB cell lines show low expression of Aurora B and MYC (Supplementary Fig. S2A). However, the cells can be modified to stably overexpress MYC by retroviral transduction[25, 26] – the stable cell lines are identified here as UW228-Myc and UW426-Myc. Comparison of protein expression in MYC-overexpressing cells compared to isogenic controls showed that increased expression of MYC was associated with an increase in Aurora A and B protein levels in unsynchronized cell culture (Supplementary Fig. S2A). The cell-cycle distribution of UW228 cells did not change with MYC overexpression while a reduction in G1/G0 cells with associated increase in polyploid cells was observed in UW426-Myc cells compared to control (Supplementary Fig. S2B).

**Figure 2:**
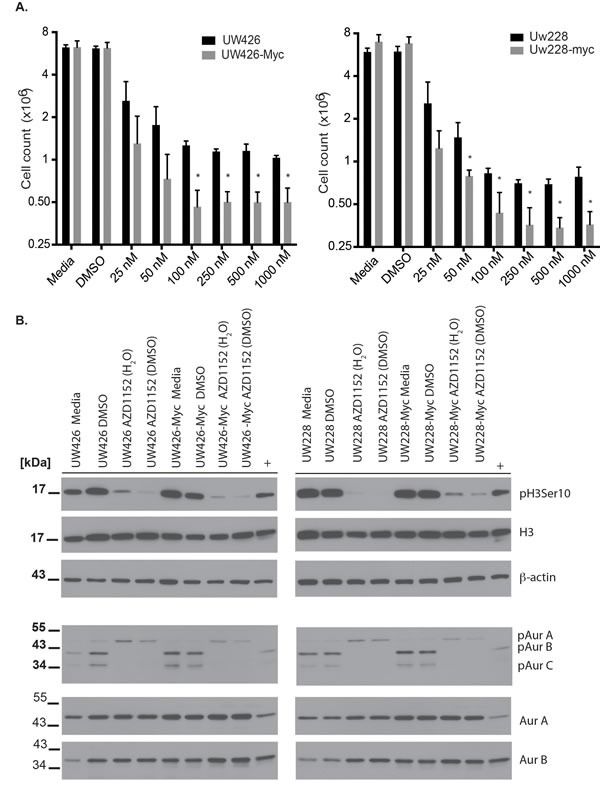
Cell viability effects and specificity of Aurora B inhibition with AZD1152-HQPA in medulloblastoma A) Cell counts obtained by Vi-Cell XR counter (Beckman-Coulter, Mississauga, Ontario) in wild-type versus MYC overexpressing medulloblastoma cells exposed for 72 hours to varying concentrations of AZD1152-HQPA. *P <0.05 for comparison between wild-type and MYC overexpressing cells. Data represent mean for three independent experiments. Error bars are standard error of the mean. A total of 1 × 10^6^ cells were cultured for 24 hours prior to exposure to AZD1152-HQPA. B) Western blots showing the inhibition of Histone H3 (Ser 10) phosphorylation and Aurora B autophosphorylation by AZD1152-HQPA at 100 nM in MYC overexpressing or wild-type cells when the drug is solubilized in water or DMSO. Labels as follows: phosphohistone H3 Serine 10 (pH3 Ser 10), histone H3 (H3), aurora kinase A (AurA), aurora kinase B (AurB), Aurora A phosphothreonine 288 (pAurA), Aurora B phosphothreonine 232 (pAurB), Aurora C phosphothreonine 198 (pAurC), β-Actin (Actin). Whole cell lysate from untreated U251 GBM cells was used as a reference antibody positive control (+). Total protein loaded was 30 μg.

The co-expression of Aurora B and MYC in MB cells suggested that Aurora B activity could be important for cell survival in the presence of excess MYC, as is the case for B-cell lymphoma cells.[15] Therefore, we tested the ability of Aurora B inhibition to elicit cell death in MYC overexpressing MB cells versus isogenic controls. We selected the Aurora B inhibitor AZD1152-HQPA for our studies as we had previously demonstrated that it had activity in brain and flank xenografts of human glioblastoma cells.[4] A series of drug concentrations was used to assess the lowest concentration of drug that could inhibit MB cell proliferation in UW228 and UW426 cells (Fig. 2A). Cell proliferation was inhibited in a concentration-dependent manner both in isogenic controls and MYC overexpressing cells (Fig. 2A). UW426 cells showed almost complete inhibition of proliferation when exposed to 100 nM AZD1152-HQPA for 72 hrs, while UW426-Myc cells had cell counts that were almost half of those at baseline, indicating that cell loss had occurred (P< 0.001, N=3). A similar effect is observed for UW228 and UW228-Myc cells, indicating that MYC overexpression sensitized cells to induction of cell death upon Aurora B inhibition (Fig. 2A). Inhibition of proliferation was achieved with 25 nM AZD1152-HQPA in UW426 and UW426-Myc as well as UW228 and UW228-Myc cells indicating a potent block of mitosis by Aurora B inhibition. The 72-hour time point was selected for determination of the concentration effects of AZD1152-HQPA based on previous observations that induction of cell death in response to Aurora B inhibition is a time-dependent phenomenon.[4]

In order to demonstrate the specificity of inhibition of MB cells at 100 nM AZD1152-HQPA, cell lysates were probed for reduction in phosphorylation of Histone H3 Serine 10, a specific substrate for Aurora B.[27, 28] Histone H3 Serine 10 phosphorylation is reduced with exposure to 100 nM AZD1152-HQPA over 24 hours without a change in total Histone H3 or β-actin protein levels (Fig. 2B). To further assess if AZD1152-HQPA could be interacting with Aurora A, a previously reported therapeutic target for MB,[29] we examined the inhibition of Aurora A and B autophosphorylation. Autophosphorylation of Aurora A on T288 and Aurora B on T232 is a key requirement for kinase activity.[30, 31] Inhibition of Aurora B autophosphorylation without a change in total Aurora B levels was achieved upon exposure of control and MYC-overexpressing cells to 100 nM AZD1152-HQPA for 24 hr (Fig. 2B). We note a weak phosphorylation signal of Aurora C (35 kDa) in UW425 and UW228 cells, which is abolished with AZD1152-HQPA (Fig 2B). The autophosphorylation of Aurora A was observed to increase slightly in both wild-type and MYC-overexpressing cells with no change in total Aurora A levels (Fig. 2B).

**Figure 3:**
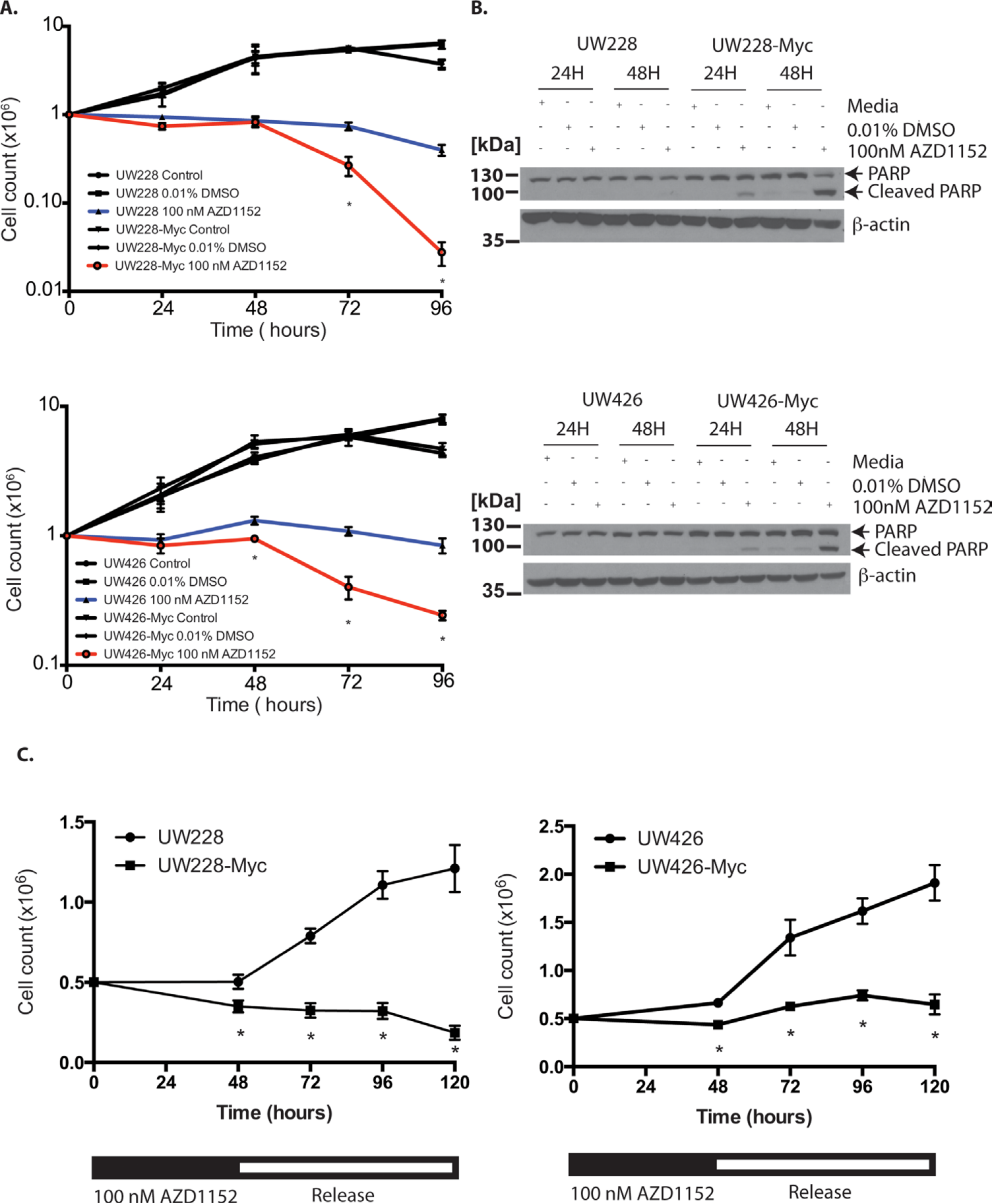
Sensitization of Myc overexpressing medulloblastoma cells to cell death and impaired cell proliferation in response to Aurora B inhibition A) Cell counts for wild-type and MYC overexpressing medulloblastoma cells treated with continuous 100 nM AZD1152-HQPA exposure for up to 96 hours. *P <0.05 for comparison between vehicle and AZD1152-HQPA treated Myc overexpressing cells. Data represent mean for three independent experiments. Error bars are standard error of the mean. B) Western blots for PARP cleavage in response to 48 hours of sustained Aurora B inhibition in Myc overexpressing medulloblastoma cells versus control. 30 μg total protein loaded. C) Cell counts in MYC overexpressing or wild-type cells exposed to 100 nM AZD1152-HQPA for 48 hours followed by drug withdrawal. Data represent mean for three independent experiments. Error bars are standard error of the mean. *P<0.05 for comparison of MYC versus wild-type cells.

The onset of cell loss in MYC-overexpressing MB cells compared to isogenic controls took place after 48 hours of continuous exposure to 100 nM AZD1152-HQPA in our time-course experiments (Fig. 3A). This cell loss was associated with PARP-1 cleavage indicating the activation of apoptosis pathways[32] preferentially in Myc overexpressing cells (Fig. 3B). While UW228 and UW426 cells showed recovery of proliferation after release from 48 hr of Aurora B inhibition, proliferation was impaired in UW426-Myc and UW228-Myc cells after release, suggesting that Aurora B inhibition has unique anti-proliferative effects on Myc overexpressing cells (Fig. 3C).

Downregulation of the serine/threonine kinase Ark5/Nuak1 has been shown to promote cell death in MYC expressing cells.[33] Furthermore, LATS1 levels are regulated by Ark5/Nuak1 phosphorylation and LATS1 is needed for activation of Aurora B.[34, 35] In order to confirm the specificity of AZD1152-HQPA action on Aurora B independent of Ark5/Nuak1, we probed for changes in LATS1 levels upon continuous medulloblastoma cell exposure to AZD1152-HQPA for up to 96 hours. We observed that LATS1 levels are preserved in the presence of 100 nM AZD1152-HQPA over the first 24-48 hours. A slight reduction in LATS1 protein is seen at 72-96 hours in both the wild-type and Myc-overexpressing cell lines. (Supplementary Fig. S3) These results indicate that the reduction of Aurora B kinase activity and tumor cell polyploidy observed in the first 24-48 hours of Aurora B inhibition with AZD1152-HQPA is independent of Ark5/Nuak1 kinase regulated LATS1 levels.

**Figure 4:**
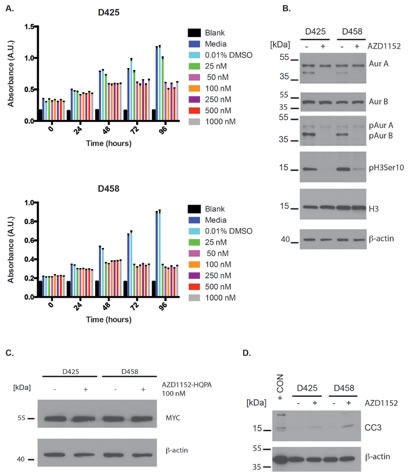
Anti-proliferative and pro-apoptotic effects of Aurora B inhibition in Group 3 medulloblastoma cells A) MTS cell viability assay of D425 and D458 cells exposed to varying concentration of AZD1152-HQPA over a 96 hour period. Error bars represent standard error of the mean of 8 replicates per group per time point. B) Western blots showing the inhibition of Histone H3 (Ser 10) phosphorylation and Aurora B autophosphorylation by AZD1152-HQPA at 100 nM in D425 and D458 endogenous MYC overexpressing cells. Labels as follows: phosphohistone H3 Serine 10 (pH3 Ser 10), histone H3 (H3), aurora kinase A (AurA), aurora kinase B (AurB), Aurora A phosphothreonine 288 (pAurA), Aurora B phosphothreonine 232 (pAurB). Total protein loaded 30 μg. Note the lower band observed in the AurA blot is non-specific binding after re-probing of the membrane. C) Western blot for MYC in D425 and D458 cells exposed to 0.01% DMSO or 100 nM AZD1152-HQPA for 48 hours. Total protein loaded 30 μg. D) Western blots for cleaved caspase-3 (CC3) in D425 and D458 cells exposed to 0.01% DMSO or 100 nM AZD1152-HQPA for 48 hours. Positive control (+ CON) was 10 μL of etoposide treated Jurkat cell lysate (Cell Signaling).

In addition to UW228-Myc and UW426-Myc cells, we also examined effects of Aurora B inhibition in MB cell lines with endogeneous Myc overexpression. We observed a block in cell proliferation using an MTS assay over a period of 96 hr in D425 and D458 cells at AZD1152-HQPA concentrations between 25 nM and 1000 nM (Fig. 4A) Specific inhibition of Aurora B was also observed in D458 and D425 cells with 100 nM AZD1152-HQPA (Fig. 4B). These D458 and D425 cells did not show an Aurora C phosphosignal. Aurora A and B levels remained stable after 48 hr of Aurora B inhibitor exposure (Fig. 4B). Also, no change in MYC protein level was observed as a result of Aurora B inhibition (Fig. 4C). The block in cell proliferation was associated with Caspase 3 cleavage upon continuous exposure to 100 nM AZD1152-HQPA for 48 hr (Fig. 4D).

In addition to the demonstration of a biochemical response that is concordant with specific Aurora B inhibition, we observed effects on cellular morphology and DNA content characteristic of Aurora B inhibition. Both wild-type and MYC overexpressing cells showed the development of a large cell, multinucleated phenotype after 48 hr exposure to 100 nM AZD1152-HQPA (Supplementary Fig. S4A). Cells that overexpressed MYC had greater DNA content compared to wild-type isogenic controls when cytokinesis was blocked by Aurora B inhibition (Supplementary Fig. S4B and S5). Induction of endoreplication was associated with an increase in the proportion of sub-G_0_ (apoptotic) cells only in MYC overexpressing cells after 48 hr of Aurora B inhibition (Supplementary Fig. S5).

### Cell death effect of Aurora B inhibition in medulloblastoma does not require endoreplication

**Figure 5:**
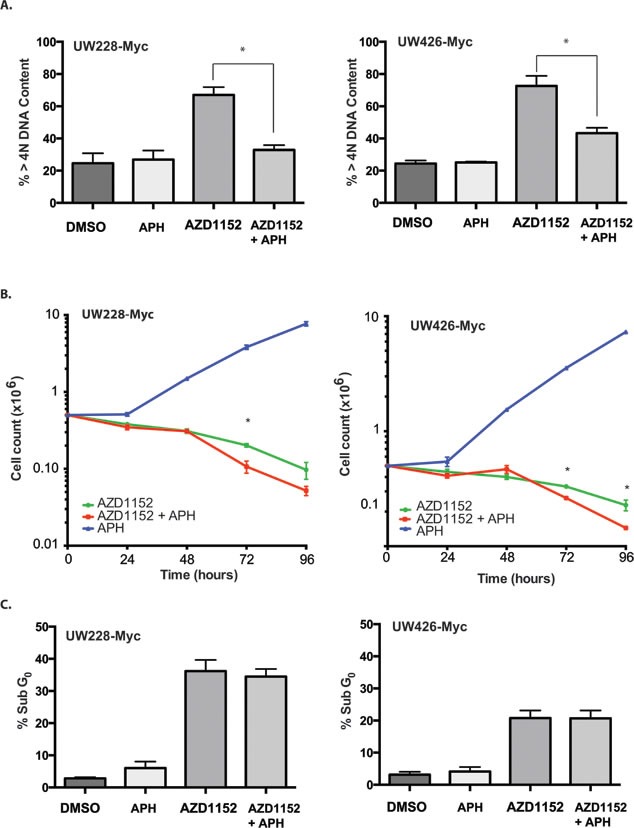
Effect of DNA polymerase inhibition on cell viability in Myc overexpressing cells subjected to Aurora B inhibition A) Percentage of cells with greater than 4N DNA content determined by FACS in MYC overexpressing cells after 24 hr of exposure to 0.01% DMSO (DMSO), 0.5 μM aphidicolin (APH), 100 nM AZD1152-HQPA (AZD1152), or 100 nM AZD1152-HQPA and 0.5 μM aphidicolin (AZD1152 + APH). *P<0.05. B) Cell counts in MYC ovexpressing cells continuously treated for 96 hr with 100 nM AZD1152-HQPA with or without exposure to 0.5 μM aphidicolin (APH) for the first 24 hr. Cells not treated with AZD1152-HQPA but exposed to aphidicolin show return of proliferation upon removal of aphidicolin after 24 hr. *P<0.05 for comparison between cells with and without inhibition of DNA replication by aphidicolin over the first 24 hr. C) Percentage of cells with subG_0_ DNA content determined by FACS in MYC overexpressing cells after 48 hr of continuous exposure 100 nM AZD1152-HQPA in the absence (AZD1152) or presence of 0.5 μM aphidicolin (AZD1152 + APH) for the first 24 hr. The graph also shows the proportion of subG_0_ cells when MYC overexpressing cells are exposed for 48 hr to control media containing 0.01% DMSO in the absence (DMSO) or presence of 0.5 μM aphidicolin (APH) for the first 24 hr.

Multiple studies have suggested that endoreplication is essential for the apoptosis response triggered by Aurora B inhibition.[16, 36-38] If this were to be the case for MB cells, this would limit the concurrent use of Aurora B inhibitors along with DNA synthesis inhibitors currently used in combination chemotherapy regimens for MB. Therefore we sought to determine if endoreplication was a requirement for cell loss in MYC overexpressing medulloblastoma. In order to address this question, the alpha-DNA polymerase inhibitor aphidicolin[39] was used to block DNA replication for 24 hr. If endoreplication is a requirement for triggering apoptosis, the expected result would be the protection of MYC overexpressing cells from the cell death induced by Aurora B inhibition. Incubation of medulloblastoma cells with 0.5 μM aphidicolin and 100 nM AZD1152-HQPA for 24 hr resulted in a significant reduction in the number of cells having > 4N DNA content (Fig. 5A). However, we did not see a protective effect of blocking endoreplication on cell viability in cells exposed to the Aurora B inhibitor (Fig. 5B,C). In fact, exposure to aphidicolin for the first 24 hours resulted in lower cell viability in both UW426-Myc and UW228-Myc cells in which Aurora B inhibition was maintained for 72 hr (Fig. 5B). In the absence of Aurora B inhibition, aphidicolin blocked cell proliferation. The release from the aphidicolin block did not affect cell viability (Fig. 5B). Interestingly, endoreplication occurred in UW426 and UW228 cells which did not demonstrate an apoptotic response to Aurora B inhibition (Fig. 3, Supplementary Fig. S5). These findings are in keeping with a DNA replication-independent trigger for apoptosis in MYC overexpressing medulloblastoma cells when subjected to Aurora B inhibition.

### Growth of MYC medulloblastoma flank xenografts is impaired by Aurora B inhibition

**Figure 6:**
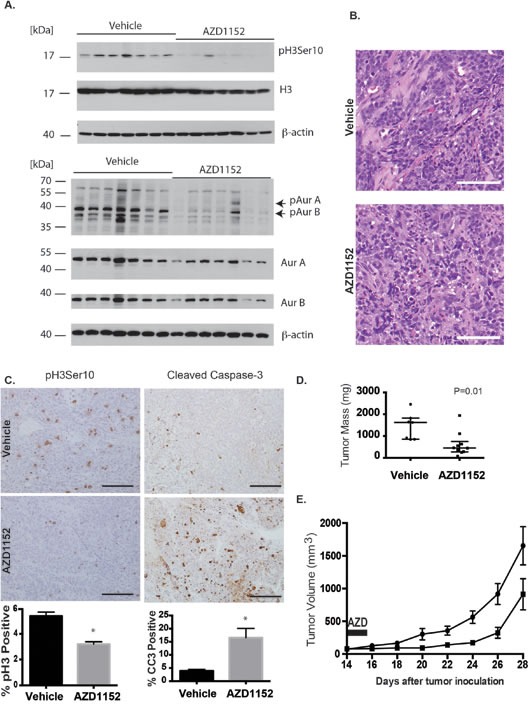
Aurora B inhibition in a Myc overexpressing medulloblastoma flank xenograft model A) Flank tumors were established in female nude mice by subcutaneous injection of UW426-Myc cells in a matrigel matrix. At 24 days post tumor cell implantation, mice were randomized to receive vehicle or 25 mg/kg/day AZD1152-HQPA x 4 days. Tumors were excised and frozen in liquid nitrogen on the 5^th^ day for Western blot analysis. Western blot shows inhibition of Aurora kinase B target Histone H3 Serine 10 (pH3 Ser10) and inhibition of autophosphorylation of Aurora B at threonine 232 (pAurB) in tumors from animals that received AZD1152-HQPA. Total protein loaded was 30 μg. B) H&E stain of flank UW426-Myc tumors in mice that received either vehicle or AZD1152-HQPA 25 mg/kg/day x 4 days. Scale bar 100 μm. C) Immunohistochemistry for Histone H3 Serine 10 phosphorylation and cleaved caspase-3 in UW426-Myc flank tumors from mice treated with vehicle (n=7) or AZD1152-HQPA (n=5). Graphs represent cell counts in more than 1000 cells per tumor per condition, *P<0.05. Scale bars 100 μm. D) Flank tumor mass on day 29 after tumor cell implantation in animals that received 50 mg/kg twice daily for 2 days starting on day 14 after cell implantation. Median and interquartile range depicted (Vehicle, n=7; AZD1152, n=10). E) Flank tumor volume in mice treated with vehicle (circles, n=6) or AZD1152-HQPA (squares, n=10) at 50 mg/kg day twice daily for 2 days starting on day 14 after tumor cell implantation. Tumor volumes are significantly different (P<0.05) from 4 days after treatment onwards.

To test the results of Aurora B inhibition in-vivo, we studied the drug-target effects using whole tumor lysates probed for Histone H3 Serine 10 phosphorylation and Aurora B autophosphorylation after administering 25 mg/kg/day of inhibitor subcutaneously for 5 days (Fig. 6A). The cells in tissue sections from UW426-Myc tumors exposed to AZD1152-HQPA were multinucleated and had lost the nodular pattern of cell organization observed in untreated tumors (Fig. 6B). The proportion of phosphohistone H3 (Ser10) positive cells was lower in the flank tumors from drug treated mice compared to vehicle controls (Fig. 6C). Furthermore, the proportion of cleaved Caspase-3 positive cells was higher in mice with UW426-Myc flank tumors that were treated with AZD1152-HQPA compared to vehicle controls (Fig. 6C). Having confirmed target activity of AZD1152-HQPA for medulloblastoma flank tumors, the growth rate of the tumors was studied by measuring tumor volume. A significant reduction in UW426-Myc tumor volume and weight at 28 days after tumor cell implantation was achieved with subcutaneous administration of 50 mg/kg twice daily for 2 days (Fig. 6D). This high dose, short course therapy was tested based on the observations that at least 48 hr of Aurora B inhibition was required to trigger cell death in UW426-Myc cells.

### Aurora B inhibition impairs cerebellar MYC medulloblastoma xenograft growth and prolongs survival

**Figure 7:**
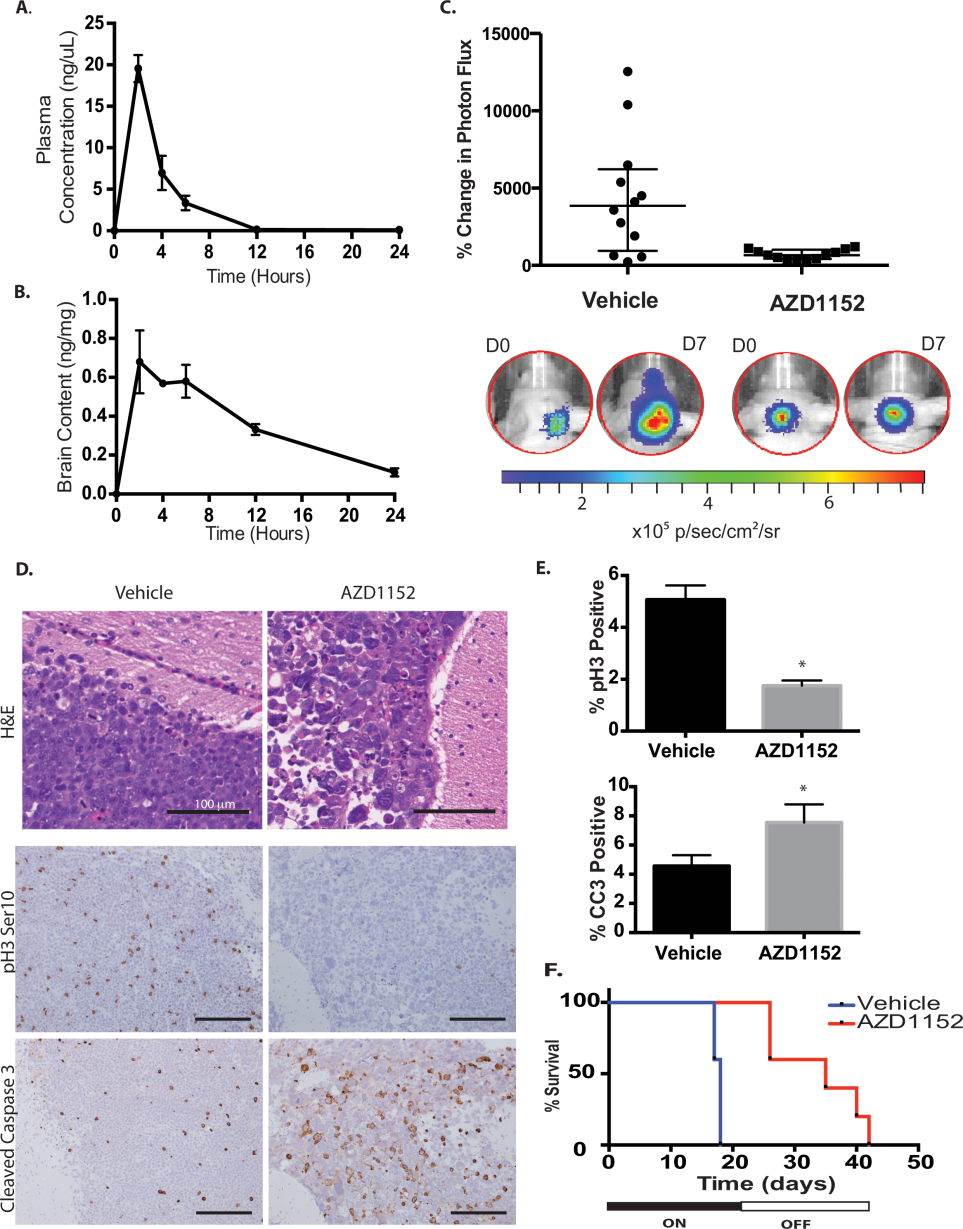
Aurora B inhibition in D458 human medulloblastoma intracranial xenograft model A) Plasma concentration of AZD1152-HQPA in mice measured by LC/MS/MS at different time points after administration of 100 mg/kg subcutaneously in the dorsal skin fold. Error bars represent S.E.M. B) Whole brain content of AZD1152-HQPA in non-tumor bearing mice measured by LC/MS/MS at different time points after subcutaneous administration of 100 mg/kg (~3 mg per animal). Error bars represent S.E.M. C) Bioluminescence measurements represented as a change in photon flux from intracranial tumor at 1 week compared to start of treatment in mice treated with vehicle (n=12) or AZD1152-HQPA 50 mg/kg/day (n=12), P<0.01. D) H&E stain and immunohistochemistry for Histone H3 Serine 10 phosphorylation (pH3 Ser10) and cleaved caspase-3 (CC3) on cerebellar D458 tumors in mice that received either vehicle (n=6) or AZD1152-HQPA 50 mg/kg/day x 7 days (n=7). Scale bars 100 μm. E) Percentage of cells positive for pH3Ser10 or cleaved caspase-3 in cerebellar D458 tumors from mice that received either vehicle (n=6) or AZD1152-HQPA 50 mg/kg/day x 7 days (n=7), *P<0.05. F) Survival curve for mice bearing D458 cerebellar tumors treated with vehicle (n=5) or AZD1152-HQPA (n=5) 50 mg/kg/day for 21 days beginning 7 days after tumor cell implantation, Log-Rank P=0.003.

The pharmacokinetic parameters for subcutaneous administration of AZD1152-HQPA in nude mice using a one-component model were as follows: *k* = 0.24 hr^−1^; *V* = 190 μL; C_0_ = 13.3 ng/μL; t_1/2_ = 2.9 hours; AUC_linear_ = 68 ng • hours/μL (Fig. 7A). The calculated effective therapeutic plasma concentration time was 11 hr for a dose of 2.5 mg (equivalent to 50 mg/kg for a 25 gm mouse). The biodistribution of AZD1152-HQPA in the brain was confirmed using LC/MS/MS after subcutaneous administration of the drug in a phosphate buffered saline solution. The peak brain content of AZD1152-HQPA was 0.7 ± 0.2 ng/mg brain tissue (n=4) at 2 hr after administration.

The D458 cell line was modified to express Luciferase by lentiviral transduction (D458-Luc/GFP). We observed the formation of D458-Luc/GFP tumor cell grafts in the cerebellum and monitored growth by bioluminescence imaging (BLI) over time. Daily administration of AZD1152-HQPA 50 mg/kg subcutaneously for a 21-day period resulted in impaired tumor growth as measured by percent change in photon flux on BLI on day 7 of therapy (Fig. 7B). Tumor cells in the cerebellum and subarachnoid spaces from mice that received AZD1152-HQPA showed large multinucleated cells on H&E histology at day 7 of therapy (Fig. 7C). Furthermore, the proportion of phosphohistone H3 (Ser10) positive cells decreased and the proportion of cleaved-caspase 3 positive tumor cells increased with drug treatment (Fig. 7C, D). In addition to a reduction in tumor growth we observed an increase in mean survival of mice bearing intracranial D458 tumors from 18 ± 0 days in the vehicle treated group to 34 ± 3 days in the AZD1152-HQPA group (Log Rank P= 0.003, Fig. 7E). All of the drug treated animals died after withdrawal of therapy. BLI demonstrated tumor growth (5/5) and metastasis (3/5) after cessation of drug therapy. Analysis of H&E spinal cord sections revealed micrometastasis in 3/4 vehicle treated mice and 4/5 AZD1152-HQPA treated mice on day 7 of treatment, suggesting that Aurora B inhibition does not alter the propensity for metastasis in MYC amplified medulloblastoma cells.

## DISCUSSION

Aurora kinase family proteins A and B have unique functions in mitosis despite sharing a high similarity. In this study, we have inhibited the kinase activity of Aurora B in MB cells with both transgene mediated and endogenous overexpression of MYC. As previously reported AZD1152-HQPA inhibits both Aurora B and Aurora C autophosphorylation[4] and this was demonstrated in the UW425 and UW228 wild-type and MYC-overexpressing cells within this study. However, medulloblastoma cells D425 and D458 lack an Aurora C phosphosignal suggesting that Aurora C function is not required for mitosis in at least some medulloblastoma cells. It is important to note that Aurora C has overlapping function with Aurora B in mitosis.[40]

We show in this study that medulloblastoma cells overexpressing MYC are sensitized to apoptosis induced upon Aurora B inhibition. The induction of apoptosis in these cells is time dependent, with cell loss occurring only after 48 hours of continuous inhibitor exposure. While endoreplication takes place upon Aurora B inhibition as a result of cytokinesis block and G2/M escape and this process is driven by MYC overexpression, we found that inhibition of DNA replication does not protect against cell loss. Furthermore, endoreplication occurred in cells that did not overexpress MYC and these cells did not show an apoptotic response. These observations suggest that DNA synthesis inhibitors should not affect the therapeutic effects of Aurora B inhibition in medulloblastoma cells overexpressing MYC. This is an important consideration since medulloblastoma is often treated with multiple chemotherapeutic drugs, including inhibitors of DNA synthesis such as methotrexate and topoisomerase inhibitors.[41] A synergistic effect on blocking cell proliferation has been shown by Aurora B inhibition and concurrent inhibition of DNA synthesis with Topoisomerase II inhibitor daunorubicin in human leukemia.[42]

Contrary to our results and those of Yang *et al*.,[42] retinal pigment epithelium cells overexpressing MYC in which DNA replication is blocked by aphidicolin are protected from induction of apoptosis by VX-680, an inhibitor of both Aurora A and B.[16] It is important to note that DNA synthesis was inhibited prior to Aurora kinase inhibition in that study, which means that the cells did not enter a 4N state. We propose that the trigger for the apoptotic response to Aurora B inhibition is initiated prior to endoreplication, but requires entry into G2/M. This is in keeping with the recently reported mechanism of ATM-dependent DNA damage response activated upon telomere deprotection that results from prolongation of mitosis by mitotic inhibitors, including Aurora kinase B inhibitor Hesparadin.[43]

Prior reports of Aurora B inhibition in colon carcinoma cells with ZM447439 in which post-mitotic G1 arrest was initiated by forced expression of p21 or p27, showed that endoreplication was required for apoptosis in these cells.[37] We did not observe apoptosis in UW426 and UW228 cells despite the induction of endoreplication in these cells. In order to explain this cell-type dependent response to Aurora B inhibition and the delayed apoptotic response to Aurora B inhibition in UW426-Myc and UW228-Myc cells we propose that transcriptional changes take place that are unique to MYC-overexpressing cells and that these changes modify the cellular response to Aurora B inhibition. Previous groups have reported upregulation of pro-apoptotic genes and down-regulation of anti-apoptotic genes with Aurora B inhibition in glioblastoma and colon cancer cells.[44, 45] Medulloblastoma cells engineered to overexpress MYC by retroviral transduction showed an upregulation of ribosomal biosynthesis genes, which is in keeping with the role of MYC as a transcriptional amplifier driving pathways involved in RNA synthesis and protein production.[46] The role of transcriptional response in driving sensitization to Aurora B inhibition and its potential for therapeutic application remains to be addressed. Changes in the gene expression state of MYC-overexpressing cells could explain the observation that these cells proliferate at a slower rate than untreated cells when released from Aurora B inhibition after a period of 48 hours.

We have demonstrated the therapeutic potential of Aurora B inhibition in MYC overexpressing medulloblastoma in both a flank and an intracranial xenograft model. The flank xenograft model allowed us to assess the efficacy of AZD1152-HQPA in a location where blood brain barrier penetration does not have a bearing on the treatment of tumor cells. This model further facilitated the accurate biochemical readouts that confirm AZD1152-HQPA could effectively reach its target and initiate the appropriate downstream effects of Aurora B inhibition. The orthotopic cerebellar xenograft model demonstrated that AZD1152-HQPA can cross the blood brain barrier at effective concentrations to inhibit tumor cell growth and prolong survival.

AZD1152-HQPA has a clear cytostatic effect *in-vivo*, with regrowth of tumor upon withdrawal of drug therapy. We have demonstrated that if cells are exposed to persistent Aurora B inhibition in-vitro for at least 48 hours that the rate of cell proliferation is reduced following drug withdrawal. These effects are not mirrored in our in-vivo models and may be explained by sub-therapeutic levels of Aurora B inhibitor at the tumor site, combined with persistence of tumor cells that are not actively cycling. Pharmacokinetic analysis indicates that during treatment there were periods in which the plasma concentration of the drug was below the *in-vitro* therapeutic target of 100 nM. Given the significant suppression of tumor growth with a single 48-hour period of high-dose therapy in the UW426-Myc flank model, it is possible that sustained and efficient tumor suppression could be achieved with a short (48-72 hr) course of AZD1152-HQPA administered at a dose of 50 mg/kg every 8 hours in mice. The presence of AZD1152-HQPA in brain has been recently documented and we also confirm this finding.[47] AZD1152 which is the pro-drug for AZD1152-HQPA is the only selective Aurora B inhibitor that has been assessed in phase I and II clinical trials. In a clinical trial AZD1152 was administered as a 1200 mg infusion over 7 days every 21 days in 32 AML patients with completion of ≥ 2 cycles in 50%.[48] The geometric mean plasma half-life of AZD1152-HQPA was 77.5 hr with steady-state plasma concentration being 245.2 ng/mL (0.483 M).[48] Thus, a plasma concentration greater than 100 nM, which was the therapeutically effective concentration in our tumor model, is achievable in humans.

Our study demonstrates that intracranial medulloblastoma tumor growth can be impaired with AZD1152-HQPA. These findings provide evidence to support the investigation of Aurora B inhibitors for the treatment of primary CNS malignancy and specifically their use as a targeted therapeutic agent for G3M tumors. The use of Aurora B inhibitors for the treatment of G3M tumors may be further evaluated by examining the effectiveness and optimal dosing of of AZD1152-HQPA in murine models of G3M tumors such as those described by Pei et al.[49] and Kawauchi et al.[50]

## MATERIALS AND METHODS

### Cell culture

Medulloblastoma cells were cultured in standard media at 37°C in a 5% CO_2_ atmosphere. All cells were confirmed to be mycoplasma negative by PCR techniques at the Hospital for Sick Children microbiology laboratory. Adherent cells were passaged using 0.05% Trypsin/0.53 mM EDTA. A list of all cell lines and culture media is provided in Supplementary Methods S1.

### Bioluminescent cell line

D458 cells were transfected with lentivirus carrying an expression cassette for Luciferase and GFP (D458-Luc/GFP) as described previously.[4]

### Western Blots

Cell lysates were derived by using RIPA lysis buffer for non-phosphoproteins, or modified RIPA buffer as described previously.[4] Lysates from flank tumors were obtained by grinding the liquid nitrogen frozen whole tumor with a mortar and pestle and suspending the powder in modified RIPA lysis buffer. Proteins were separated by SDS-PAGE on 10 to 12.5% gels and transferred to PVDF membranes using a semi-dry transfer apparatus (Bio-Rad, Hercules, CA). The membranes were washed in TBS-T (100 mM Tris-Cl, pH 7.5, 150 mM NaCl, 0.1% Tween-20). Incubation with primary antibody in TBS-T with 5% Bovine Serum Albumin (Sigma) or 5% non-fat milk was performed at 4°C overnight followed by incubation with horseradish peroxidase conjugated secondary antibody for 1 hr and enhanced chemiluminescence detection (Supplementary Methods S1).

### Cell viability assays

Cell viability was assessed over 96 hr using MTS (3-(4,5-dimethylthiazol-2-yl)-5-(3-carboxymethoxyphenyl)-2-(4-sulfophenyl)-2H-tetrazolium) absorbance (Promega, Madison, WI, USA) at 490 nm with at least 8 repeats per treatment condition per time point in each experiment. For cell count experiments, 1 × 10^6^ cells were cultured on a 3-cm Petri dish for 24 hr prior to exposure to drug inhibitor and grown for a total period of 96 hr with varying concentrations of AZD1152-HQPA (Selleck Chemicals LLC, Houston, TX, USA) dissolved in dimethyl sulfoxide (DMSO, Sigma, St. Louis, MO, USA). The final DMSO concentration was 0.01% v/v in media. The live cell number was determined by trypan-blue exclusion using a Vi-Cell XR counter (Beckman-Coulter, Mississauga, Ontario). Experiments were independently repeated three times.

### Immunofluorescent labeling and imaging

Cells were washed with warm (37°C) phosphate buffered saline (PBS, Wisent Inc.) and fixed with 4% paraformaldehyde at room temperature for 10 minutes. The cell membranes were permeabilized with 0.5% v/v Triton-X100 (Sigma) in PBS. Antigens were blocked with 1% bovine serum albumin fraction V (Merck) in PBS for 1 hr at room temperature followed by immunolabeling. The primary and secondary antibodies used, incubation protocol, and DNA staining and are described in Supplementary Methods S1. An Olympus 1X81 spinning disc confocal microscope (Olympus Canada Inc., Richmond Hill, ON, Canada) with Yokogawa scan head (Yokogawa Corporation of America, Sugar Land, TX, USA) was used to visualize the cells with 20X air or 40X water immersion lenses (Carl *Zeiss* AG, Germany). Images were processed using Volocity 5.5 imaging software.

### FACS cell cycle analysis

Cells were grown to confluence, dissociated, and pelleted for resuspension in 50 μL of staining media (1M HEPES (pH 7.2), 1M NaN_3_, 2% fetal bovine serum in Hank's Buffered Salt Solution). The cell suspension was gently mixed with 1 mL 80% ethanol. The cells were centrifuged at 200 x *g* for 5 min at 4°C for pelleting and resuspended in Hank's Buffered Salt Solution (HBSS, Wisent Inc.) containing 2 mg/mL RNAse A (Qiagen Inc., Toronto, ON, Canada) for 5 minutes at room temperature. Cells were then pelleted and resuspended in HBSS containing 0.1 mg/mL propidium iodide and 0.6% (w/v) NP40 at room temperature for 30 minutes. Subsequently, the cell pellet was resuspended in 500 μL of staining media and filtered through a 100 μm nylon cell strainer (BD Biosciences Discovery Labware, Bedford, MA, USA). A total of 10,000 cells were sorted for each cell line and experimental condition and each experiment was performed in triplicate. An LSR II analyzer (Becton Dickinson, Franklin Lakes, NJ, USA) was used with FACSDiva software. The excitation wavelength was 523 nm and the emission filter was LP600, BP610/20. Data analysis was conducted with FlowJo (Tree Star Inc., Ashland, OR, USA). Doublets were excluded by gating using the PI-intensity versus FSC-W graph as described previously.[4]

### Xenograft tumor model

Animal experiments were approved by the Hospital for Sick Children Animal Care Committee (protocol 1000010458) and conducted in accordance with the Ontario Animals for Research Act and the Canadian Council for Anima Care guidelines. Athymic nude 8-10 week old female mice *Foxn1nu/Foxn1nu* (Charles River, Sherbrook, QC, Canada) were used for flank xenografts and intracranial xenografts. Isofluorane gas anaesthetic was used to induce an insensate state for inoculation of cells into the subcutaneous space at the right flank. For implantation, 2 × 10^6^ cells were suspended in a 1:1 mixture of PBS and Matrigel (Becton Dickinson). A total volume of 200 μL cell suspension was injected into the subcutaneous space using a 30 Ga needle. Fourteen days after cell inoculation palpable tumors were measured with a digital caliper to estimate the tumor volume (in mm^3^) using the formula: tumor volume = length (mm) x width^2^(mm^2^)/2. Animals were weighed for drug dosage calculation and randomized to a vehicle or drug treatment group. Subcutaneous bolus injection in the dorsal skin fold of each animal was performed daily for 4 days. AZD1152-HQPA was administered at a weight-determined volume to achieve 25 or 50 mg/kg/day using a 2 or 4 mg per mL of 3.94% v/v DMSO in PBS solution. The vehicle group received a weight-determined volume of PBS with 3.94% DMSO vehicle solution. Tumor volumes were measured every 2 days until day 30 after implantation of tumor cells. Animals were euthanized by carbon dioxide chamber either at day 5 or day 30 post-implantation and tumors excised and immediately immersed in 3.7% formaldehyde for histologic analysis or frozen in liquid nitrogen for protein analysis. Formalin-fixed whole tumors were weighed prior to sectioning

Intracranial implantation of 2.5 × 10^5^ cells suspended in 3 μL of PBS was performed using sterile technique under isofluorane gas anaesthetic. Cells were placed into the right cerebellum at a depth of 2.5 mm via a single burr hole 2 mm lateral to the midline and 1 mm posterior to the junction of the sagittal and lamdoid sutures. Mice received 5 mg/kg ketoprofen analgesic and a bolus of 0.5 mL 0.9% saline subcutaneously in the immediate post-operative period. At day 7, after implantation all mice were imaged using Luciferin bioluminescence as described previously.[4] Mice with defined posterior fossa tumors were randomized to AZD1152-HQPA or vehicle treatment. AZD1152-HQPA was given at a dose of 50 mg/kg/day using a 4 mg AZD1152-HQPA per mL of 3.94% v/v DMSO in PBS administered in the dorsal skin fold. Treatment was continued for 21-days and Luciferin bioluminescence imaging was repeated at 7, 15, and 28 days after initiation of therapy. The vehicle group received a weight-determined volume of PBS with 3.94% v/v DMSO vehicle solution. The survival endpoints were death, weight loss greater than 20%, inability to mobilize, continuous seizure, moribund state. Tumors were excised for histological analysis 7 days after initiation of drug therapy and at the time of animal death.

### Pharmacokinetic experiment and AZD1152-HQPA quantification

Mice were injected subcutaneously in the dorsal skin fold with 100 mg/kg AZD1152-HQPA using a solution of 4 mg AZD1152-HQPA per mL of 3.94% v/v DMSO in PBS. At 2 hr, 4 hr, 6 hr, 12 hr, and 24 hr after drug administration, blood was drawn by cardiac puncture and collected in EDTA tubes. The mice were perfused with heparinized saline (5 units heparin/mL 0.9% NaCl). Subsequently the brain was excised and frozen in liquid nitrogen. Blood samples were centrifuged at 800 x *g* for 10 min and the supernatant plasma was frozen in liquid nitrogen. Frozen brain and blood samples were submitted to the Analytical Facility for Bioactive Molecules, The Hospital for Sick Children for liquid chromatography-tandem mass spectrometry (LC/MS/MS) analysis to determine AZD1152-HQPA concentration. Pharmacokinetic parameters were calculated from linear regression analysis of Log_10_(plasma concentration) versus time graph assuming a one-component model.

### Immunohistochemistry

Flank tumors and whole brains were fixed and processed for immunohistochemistry as described previously.[4] Tissues were incubated with primary antibody at room temperature for 1 hr. Rabbit polyclonal anti-cleaved Caspase 3 antibody (Cell Signaling) 1:500, and anti-phospho Histone H3 (Ser 10) (Cell Signaling) 1:200 were used. TBS-T was used for washes. Detection was performed with avidin-biotin-horseradish peroxidase complex (ABC; Vector Laboratories) followed by diaminobenzidine as the chromogen. Nuclei were counterstained with hematoxylin. Slides were visualized on an Olympus 1X37 light microscope. Cells were counted in 20X fields to greater than 1000 cells per specimen.

### mRNA expression analysis in medulloblastoma tumors

The expression profiles of medulloblastoma tumors, cell lines, and normal samples were retrieved from GEO accession GSE21140. The samples were expression-profiled and assigned subgroups as previously described [20, 51] Pearson correlation was used to determine the association between *MYC* mRNA expression and *AURKA*, *AURKB*, and *AURKC* mRNA expression in medulloblastoma tumors.

### mRNA expression profiling in cell lines

Subgrouping of D283, D425, Daoy, Med 8A, ONS-76, RES262, UW228 cells in comparison to 103 medulloblastomas was performed by NanoString analysis for selected signature genes differentiating Wnt, SHH, Group 3, and Group 4 tumors as previously described.[23]

### Statistics

The PASW Statistics 18 software (SPSS Inc., Chicago, IL) or Prism 6 (GraphPad Software, Inc., CA, USA) were used for statistical analysis. Data were assessed for normality by the Kolmogorov-Smirnov test. All measures are reported as mean +/− standard error of the mean. Means were compared by independent samples Student's t-test. One-way ANOVA was used to compare mean absorbance at varying concentrations of inhibitor for the MTS cell viability assay with post-hoc LSD testing if significant differences were found between groups. For post-hoc LSD testing after ANOVA a P-value of less than 0.025 was selected as significant. In all other analysis a P-value less than 0.05 was selected for significance. Survival analysis was performed using a Kaplan-Meier plot. The Breslow (Generalized Wilcoxon) test was used for inferential analysis with a P-value less than 0.05 selected as significant.

### SUPPLEMENTARY MATERIAL, FIGURES, TABLES


