# Supplementary material for: Mechanism of action and therapeutic efficacy of Aurora kinase B inhibition in MYC overexpressing medulloblastoma
Source: Oncotarget. 2014 Dec 31;6(5):3359–74. doi: 10.18632/oncotarget.3245 (PMC4413659; doi:10.18632/oncotarget.3245)
Supplement: Supplementary file 1 [file oncotarget-06-3359-s001.pdf]

Mechanism of action and therapeutic efficacy of Aurora kinase B inhibition in MYC overexpressing medulloblastoma

Supplementary Materials & Methods

Cell culture

The UW228, UW426, UW228-Myc, and UW426-Myc cells were cultured in Alpha modified Eagle's media (Wisent Inc., St. Bruno, QC, Canada) with 10% v/v heat inactivated fetal bovine serum (FBS, Wisent). The D425 and D458 cells were grown as suspension cultures in IMEM with 20% FBS and 1% (v/v) HEPES (Wisent). D283 cells were cultured in Dulbecco's Modification of Eagle's Media (DMEM, Wisent) with 10% FBS and 1% essential amino acids. RES262 was grown in DMEM with 2% FBS. ONS-76 and Daoy cells were cultured in DMEM with 10% FBS. D341 cells were cultured in Eagle's Minimal Essential Media with 20% FBS and 1% (v/v) MEM non-essential amino acids.

Western Blots

The following antibodies and dilutions were used: anti-cleaved Caspase 3(1) (Cell Signaling Technologies, Danvers, MA, USA) 1:1000, anti-PARP(2) (Cell Signaling) 1:1000, anti-phosphohistone H3 (Ser10)(3) (Cell Signaling) 1:500, anti-Aurora B 1:1000 (Cell Signaling), anti-Aurora A phosphothreonine 288/Aurora B phosphothreonine 232/Aurora C phosphothreonine 198 1:1000 (Cell Signaling), anti-MYC(4) 1:1000 (9E10), anti-β-actin 1:1000 – 1:10000 (Cell Signaling), anti-LATS1 1:1000 (Cell Signaling). Secondary antibodies used were anti-rabbit or anti-mouse IgG conjugated to horseradish peroxidase (Cell Signaling) at a 1:1000 to 1:5000 dilution. Enhanced

chemiluminescence detection (PerkinElmer, Waltham, MA, USA) on x-ray film was used to detect antibody signal. Equal protein loading was confirmed by re-probing blots for β-actin.

Immunofluorescent labeling and imaging

Coverslips containing fixed and permeabilized cells after blocking with bovine serum were incubated with primary antibody overnight in a moisture chamber at 4°C. The following antibodies and dilutions were used for immunolabeling: anti-Aurora B kinase polyclonal rabbit IgG 1:125 (Invitrogen), anti-α-tubulin goat polyclonal IgG 1:100 (Santa Cruz Biotechnology Inc., Santa Cruz, CA, USA). Secondary antibodies were anti-Rabbit IgG Alexa 594 (Invitrogen) and anti-goat IgG Alexa 488. Phalloidin Alexa Fluor 647 1:500 (Invitrogen) was added to the secondary antibody mix to label F-Actin. Coverslips were incubated with secondary antibody in a moisture chamber at room temperature for 1 hr. After three washes in PBS, the coverslips were incubated for 5 minutes with 1 µg/mL Hoechst 33342 stain (Invitrogen) in PBS to label DNA. Coverslips were mounted on Superfrost glass slides (Thermo Fisher Scientific Inc.) with fluorescent mounting media (Dako Canada Inc., Burlington, ON, Canada).

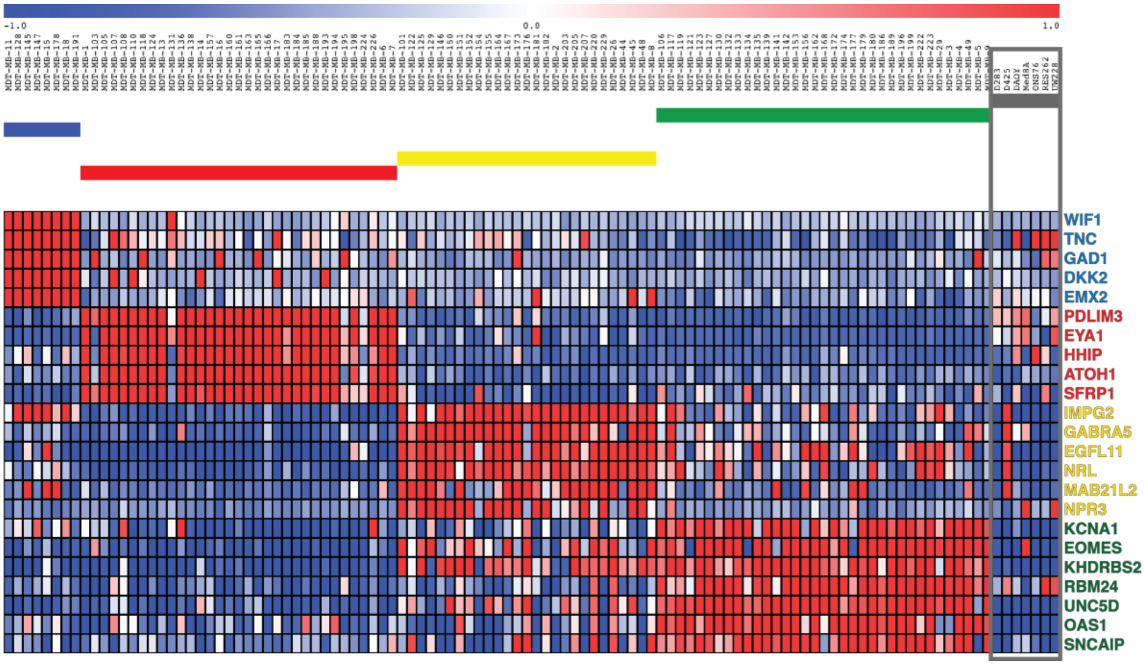

Supplementary Fig. S1: **Subgrouping of MB cell lines according to gene expression signature.** Expression status of medulloblastoma subgroup signature genes in 103 primary medulloblastomas and 7 medulloblastoma cell lines determined by NanoString assay. D425 was the only cell line tested with expression profile resembling a Group 3 signature. Genes labeled in blue are Wnt signature genes, red are SHH signature genes, yellow are Group 3 signature genes, and green are Group 4 signature genes.

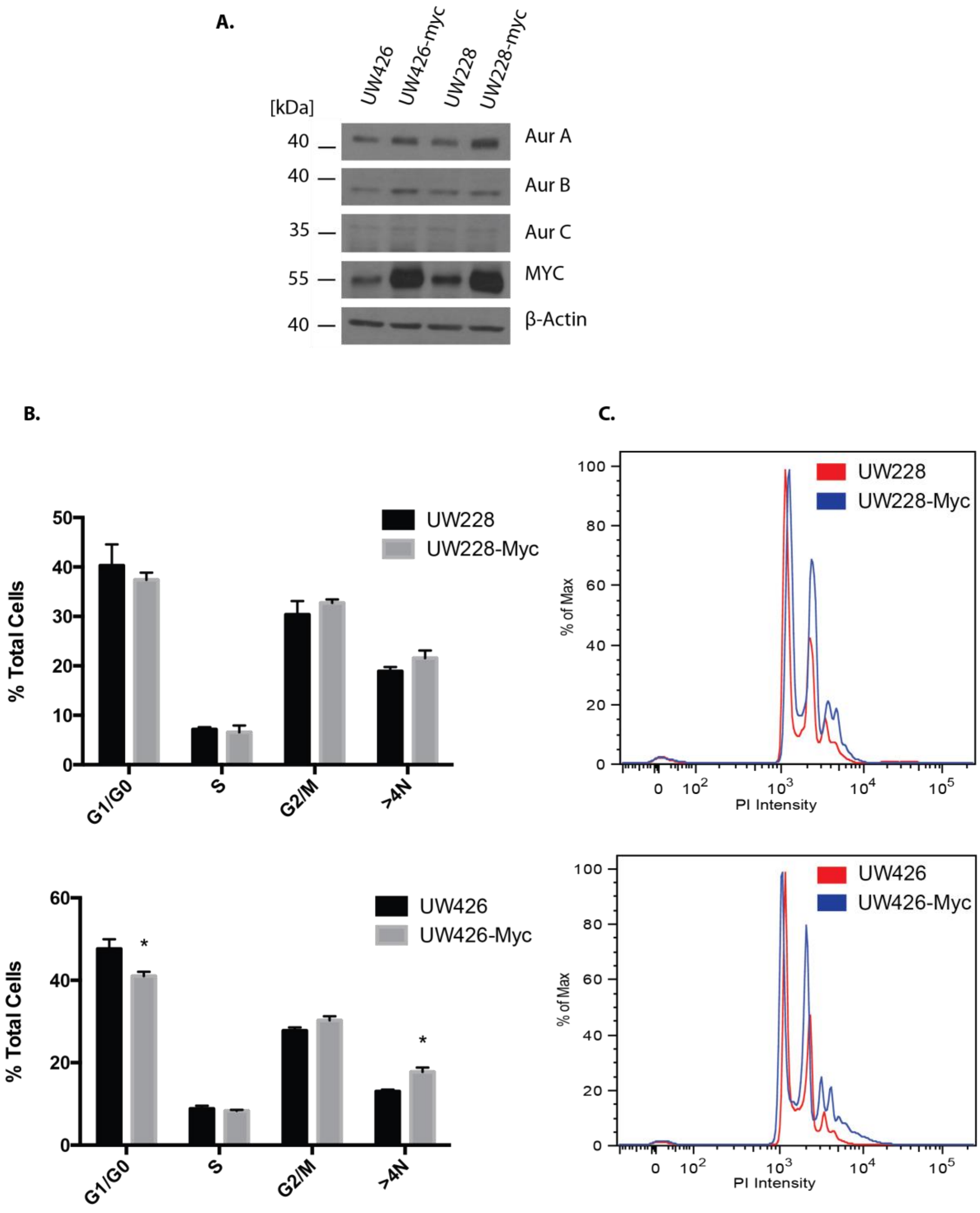

Supplementary Fig. S2: Aurora kinase expression and DNA content in Myc

overexpressing medulloblastoma cells. A) Western blot showing Aurora A, Aurora B, and Aurora C protein expression in relation to MYC protein expression in UW426 and UW228 cells upon stable overexpression of *MYC* by retroviral transduction (UW426-Myc, UW228- Myc). **B)** Analysis of DNA content as an indicator of cell-cycle status in wild-type and MYC overexpressing cells maintained in unsynchronized cell culture. Bar graphs represent cell-cycle phase gating based on propidium iodide intensity histogram from 10,000 sorted cells. * $P<0.05$, N=3 (UW228/228-Myc), N=4 (UW426/426-Myc). **C)** Representative propidium iodided intensity histograms comparing DNA content in cells from wild-type versus MYC-overexpressing medulloblastoma lines.

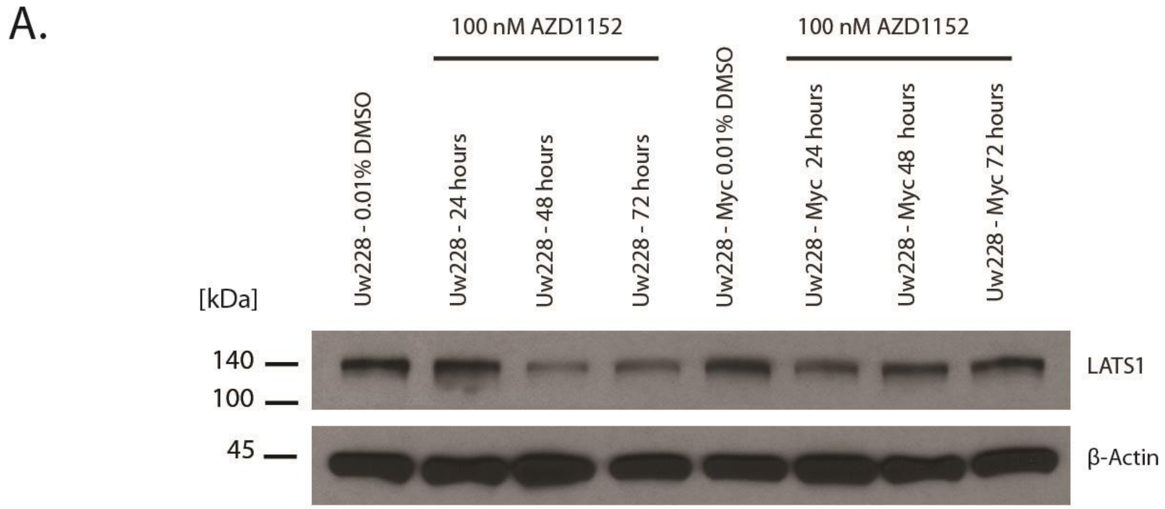

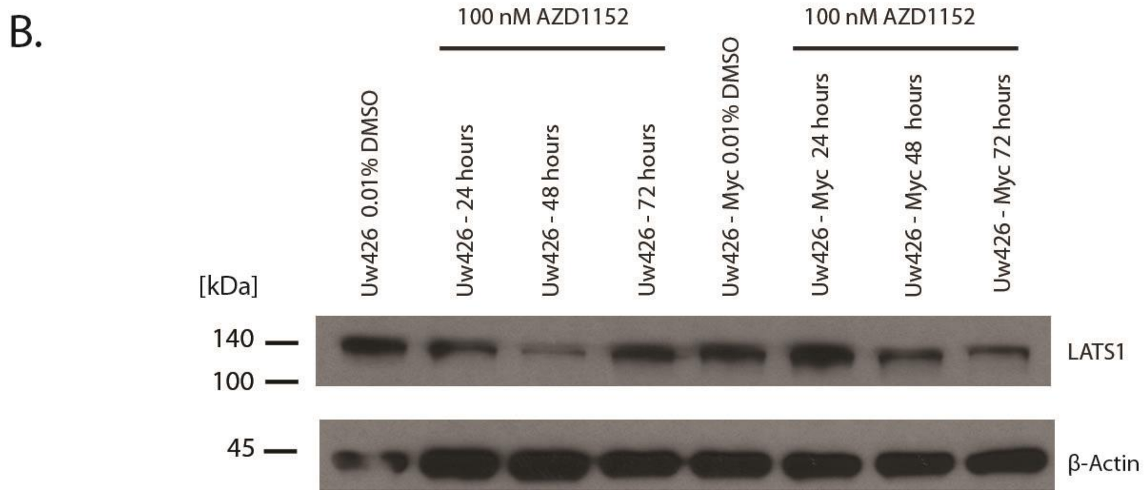

Supplementary Fig S3: Changes in LATS1 expression in response to Aurora B inhibition with AZD1152-HQPA. **A)** LATS1 protein expression was assesed by Western blot in UW228 and UW228-Myc cell lines that where treated with 0.01 % DMSO (vehicle control) or 100 nM AZD1152-HQPA. Lysates where prepared at 24, 48 and 72 hours. **B)** LATS1 protein expression was assesed by Western blot in

UW426 and UW426-Myc cell lines that where treated with 0.01 % DMSO (vehicle control) or 100 nM AZD HQPA 1152. Lysates where prepared at 24, 48 and 72 hours.

A.

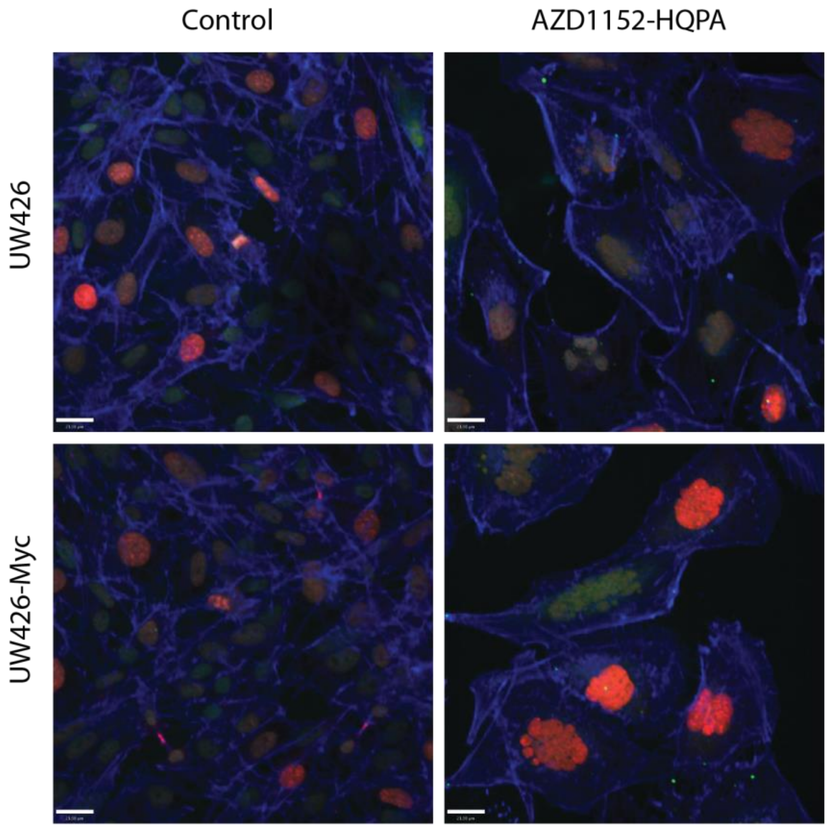
Control
AZD1152-HQPA
UW426
UW426-Myc

B.

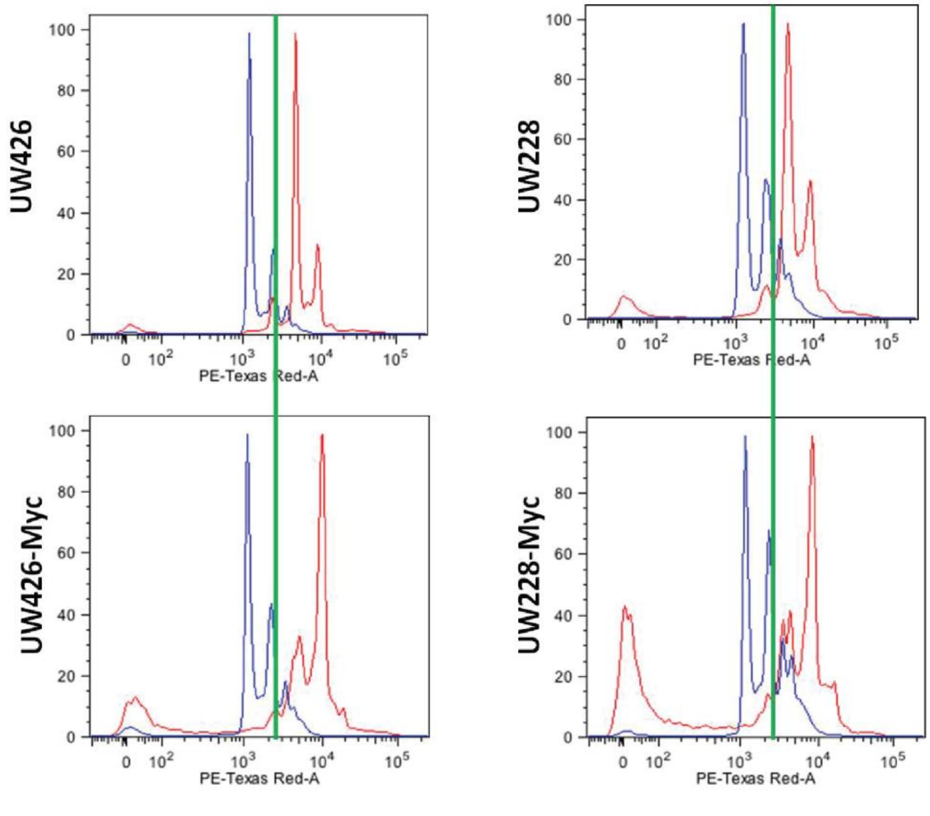
UW426
UW228
UW426-Myc
UW228-Myc
100
80
60
40
20
0
0
10^2
10^3
10^4
10^5
PE-Texas Red-A

Supplementary Figure S4: Cellular effect of Aurora B inhibition in wild-type and Myc overexpressing cells. A) Confocal fluorescence microscopy of UW426 and UW426- Myc cells exposed to 0.01% DMSO (control) or 100 nM AZD1152-HQPA for 48 hours. Red – Aurora B, Blue – F-Actin, Teal – DNA. Scale bar 24 μm. **B)** The effect of MYC overexpression on extent of endoreplication in medulloblastoma cells treated with AZD1152-HQPA for 48 hours was assessed by DNA content analysis using propidium iodide fluorescence. MYC-overexpressing cells (UW426-Myc, UW228-Myc) had greater DNA content after 48 hours of Aurora B inhibition compared to their wild-type controls (UW426, UW228). Blue – 0.01% DMSO, Red- 100 nM AZD1152-HQPA.

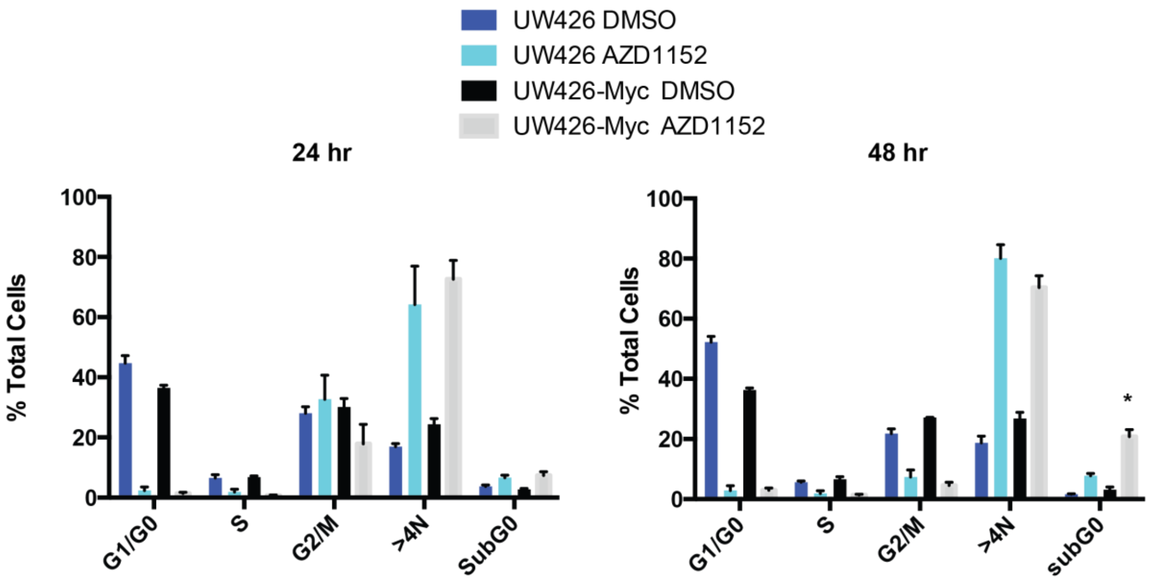
UW426 DMSO
UW426 AZD1152
UW426-Myc DMSO
UW426-Myc AZD1152
24 hr
48 hr
% Total Cells
100
80
60
40
20
0
G1/G0
S
G2/M
>4N
SubG0
subG0
*

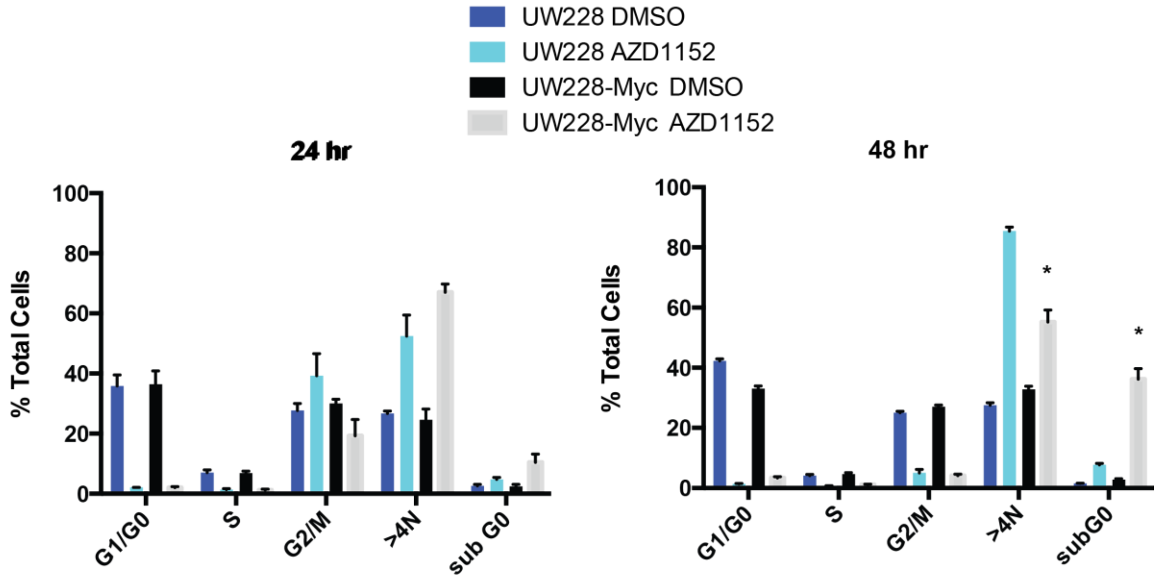
UW228 DMSO
UW228 AZD1152
UW228-Myc DMSO
UW228-Myc AZD1152
24 hr
48 hr
% Total Cells
100
80
60
40
20
0
G1/G0
S
G2/M
>4N
sub G0
subG0
*
*

Supplementary Figure S5: Effect of Aurora B inhibition on DNA content in wild-type and Myc overexpressing cells. Quantification of FACS DNA content analysis on UW426, UW228, UW426-Myc and UW228-Myc cells exposed to 0.01% DMSO or 100 nM AZD1152-HQPA for 24 or 48 hours. Data are means from three independent experiments. Error bars represent standard error of the mean. * P<0.01 for WT vs Myc in the setting of AZD1152-HQPA exposure.
